# Development of nomograms to predict recurrence after conversion hepatectomy for hepatocellular carcinoma previously treated with transarterial interventional therapy

**DOI:** 10.1186/s40001-023-01310-4

**Published:** 2023-09-09

**Authors:** Min Deng, Rongce Zhao, Renguo Guan, Shaohua Li, Zhijun Zuo, Wenping Lin, Wei Wei, Rongping Guo

**Affiliations:** 1 Department of Liver Surgery, Sun Yat-sen University Cancer Center, 651 Dongfeng East Road, Guangzhou, China; 2 State Key Laboratory of Oncology in South China, Collaborative Innovation Center for Cancer Medicine, 651 Dongfeng East Road, Guangzhou, China

**Keywords:** Hepatocellular carcinoma, Conversion, Hepatectomy, Recurrence, Nomogram

## Abstract

**Background:**

Lack of opportunity for radical surgery and postoperative tumor recurrence are challenges for surgeons and hepatocellular carcinoma (HCC) patients. This study aimed to develop nomograms to predict recurrence risk and recurrence-free survival (RFS) probability after conversion hepatectomy for patients previously receiving transarterial interventional therapy.

**Methods:**

In total, 261 HCC patients who underwent conversion liver resection and previously received transarterial interventional therapy were retrospectively enrolled. Nomograms to predict recurrence risk and RFS were developed, with discriminative ability and calibration evaluated by C-statistics, calibration plots, and the Area under the Receiver Operator Characteristic (AUROC) curves.

**Results:**

Univariate/multivariable logistic regression and Cox regression analyses were used to identify predictive factors for recurrence risk and RFS, respectively. The following factors were selected as predictive of recurrence: age, tumor number, microvascular invasion (MVI) grade, preoperative alpha‐fetoprotein (AFP), preoperative carbohydrate antigen 19-9 (CA19-9), and Eastern Cooperative Oncology Group performance score (ECOG PS). Similarly, age, tumor number, postoperative AFP, postoperative protein induced by vitamin K absence or antagonist-II (PIVKA-II), and ECOG PS were incorporated for the prediction of RFS. The discriminative ability and calibration of the nomograms revealed good predictive ability. Calibration plots showed good agreement between the nomogram predictions of recurrence and RFS and the actual observations.

**Conclusions:**

A pair of reliable nomograms was developed to predict recurrence and RFS in HCC patients after conversion resection who previously received transarterial interventional therapy. These predictive models can be used as guidance for clinicians to help with treatment strategies.

**Supplementary Information:**

The online version contains supplementary material available at 10.1186/s40001-023-01310-4.

## Background

Hepatocellular carcinoma (HCC) is the fourth most common cause of cancer-related death among malignant tumors and has a poor prognosis worldwide [1]. Overall, HCC has high morbidity, with approximately 841,000 new cases and 782,000 deaths annually in 2018 [2]. Therefore, effective treatment is of great significance for these patients. Although radical surgery is an important strategy for the treatment of HCC, approximately 80% of patients have unfortunately lost the chance to receive surgery due to a large tumor, multiple nodules, or vascular invasion at the first visit [2, 3]. Moreover, despite surgical resection in 20% of HCC patients, the postoperative recurrence rate is high, at 53–71% at 5 years after the operation [4, 5].

In general, a tumor downstaging strategy, constituting a promising research direction to convert cases not suitable for surgery to cases that can receive radical resection through various treatments, such as interventional therapies, is crucial for improving the prognosis of HCC patients [6, 7]. Nevertheless, HCC patients who undergo conversion hepatectomy also face the potential of tumor recurrence [8, 9]. To date, there are a few studies on recurrence in HCC patients who are treated with conversion hepatectomy and who previously received transarterial interventional therapy. Reliable prognostic information in HCC after hepatectomy is vital for patients and clinicians, as accurate predictive systems for HCC contribute to decision making among medical practitioners for adjuvant treatment and follow-up frequency. In addition, such knowledge is important for providing patients and their families with helpful information about treatment modalities and outcomes, especially for those with high-risk factors for recurrence.

In recent years, the development of targeted therapy, immune checkpoint inhibitors, and interventional treatment has had a significant impact on the prognosis of patients with advanced HCC and recurrent HCC [2, 10]. Therefore, for patients with a high risk of recurrence, a combination of the above treatments preoperatively or postoperatively may enable these patients to achieve a better survival outcome.

Considering the high risk of recurrence after liver resection, it is critical to detect recurrence early and to determine which patients may benefit from adjuvant or neoadjuvant therapy. Due to the lack of a specific and practical predictive method, it is necessary to establish a predictive model that integrates factors related to recurrence according to perioperative clinicopathological parameters. To this end, nomograms are easy to use, providing personalized and highly accurate risk estimation of all available models and guiding clinical treatment decisions [11].

In this study, we retrospectively analyzed recurrence in HCC patients who underwent transformation surgery and developed nomograms of recurrence risk and recurrence-free survival (RFS) probability.

## Methods

### Patients

Data for HCC patients who underwent conversion hepatectomy and previously received transarterial interventional therapy from June 2015 to June 2020 were retrospectively collected at our cancer center.

The inclusion criteria were as follows: (1) not eligible for liver resection when the initial diagnosis was made according to imaging findings, such as insufficient remaining liver volume or inability to undergo radical resection due to a large tumor, multiple nodules, or macrovascular invasion; (2) tumor response of a complete or partial response after transarterial interventional therapy; (3) R0 tumor resection; and (4) pathological confirmation of HCC. Patients who had a history of other cancers, distant metastasis that occurred by the first visit, and incomplete clinical data were excluded. This work has been reported in line with the STARD criteria [12]. This study was approved by the Institutional Review Board of Sun Yat-sen University Cancer Center (SYSUCC, Guangzhou, China) and was performed following the Declaration of Helsinki of 1975 as revised in 1983.

### Transarterial interventional therapy and evaluation

Transarterial interventional therapy includes transcatheter arterial chemoembolization (TACE), hepatic artery infusion chemotherapy (HAIC), and TACE combined with HAIC. The relevant protocol involved TACE with 50 mg of lobaplatin, 50 mg of epirubicin, and lipiodol and HAIC with the mFOLFOX regimen (oxaliplatin 85 mg/m^2^, leucovorin 400 mg/m^2^, fluorouracil bolus 400 mg/m^2^ on day 1 and fluorouracil infusion 2400 mg/m^2^ for 46 h, every 3 weeks).

In the TACE group, we evaluated patient responses after 4 weeks of treatment; in the HAIC group or TACE combined with HAIC group, we assessed responses after two cycles of treatment. Computed tomography (CT) or magnetic resonance imaging (MRI) was used for evaluation. The maximum treatment cycle is no more than six cycles. Tumor response was evaluated by the modified Response Evaluation Criteria in Solid Tumors (mRECIST [13]), including the following types: (I) complete response (CR), (II) partial response (PR), (III) stable disease (SD), and (IV) progressive disease (PD). Surgical resection is considered when PR or CR is reached.

### Liver resection and follow-up

Liver resection was performed when preoperative assessments, such as liver and renal function tests, cardiopulmonary function tests, liver cancer-specific tumor marker analysis, and abdominal imaging, were completed.

Patients were followed up once every month after surgery; examinations included routine blood tests, liver function tests, tumor markers, and abdominal imaging (ultrasonography, CT, or MRI) at each follow-up visit. The diagnostic criteria for tumor recurrence were as follows: (1) the appearance of new lesions with typical radiologic features of HCC on two imaging studies and (2) evidence of new extrahepatic lesions not identified preoperatively. The endpoints of this research were tumor recurrence and time to recurrence. RFS was calculated from the date of hepatectomy to the date when tumor recurrence was diagnosed or the date of the last follow-up visit.

### Statistical analysis

Continuous variables are expressed as the median and interquartile range (IQR). Categorical data are expressed as numbers (percentages). Time to recurrence or censoring was estimated using the Kaplan–Meier method, and differences were compared using the log-rank test. All statistical tests were 2 tailed. Univariate logistic regression analyses were performed to predict the probability of tumor recurrence, and multivariable logistic regression analyses were carried out on variables that reached *p* < 0.2 in univariate analysis. The final multivariate regression model was built from the set of candidate variables by removing predictors based on p-values in a stepwise manner. Similarly, variables with *p* < 0.2 in univariate analysis were subjected to multivariate analysis using stepwise selection in a Cox regression model to identify predictive factors for RFS at 2 and 3 years. Statistically significant factors (*p* < 0.05) from multivariate analysis were entered into nomograms.

The bootstrap resampling method was carried out for internal validation of the predictive models, selecting 1000 repetitions. For each group of 1000 bootstrap samples, the model was refitted and tested against the observed sample to estimate the predictive accuracy and bias. The predictive accuracy of the models was measured using the concordance index (C-index) and calibration quantifying the level of agreement between the predicted probabilities and the actual possibility of having the event of interest. Model discrimination was assessed by calculating the area under the receiver operating characteristic (AUROC) curve (or C-statistic). Model calibration was determined by the Hosmer–Lemeshow (H–L) technique, and a calibration curve was drawn. The statistical analyses were performed using IBM SPSS Statistics (version 26.0, SPSS Inc., Chicago, IL, USA), and the nomograms were developed using R software (version 4.0.2, Regression Modeling Strategies package. The R Foundation for Statistical Computing, Vienna, Austria).

## Results

### Clinicopathologic features

**Fig. 1:**
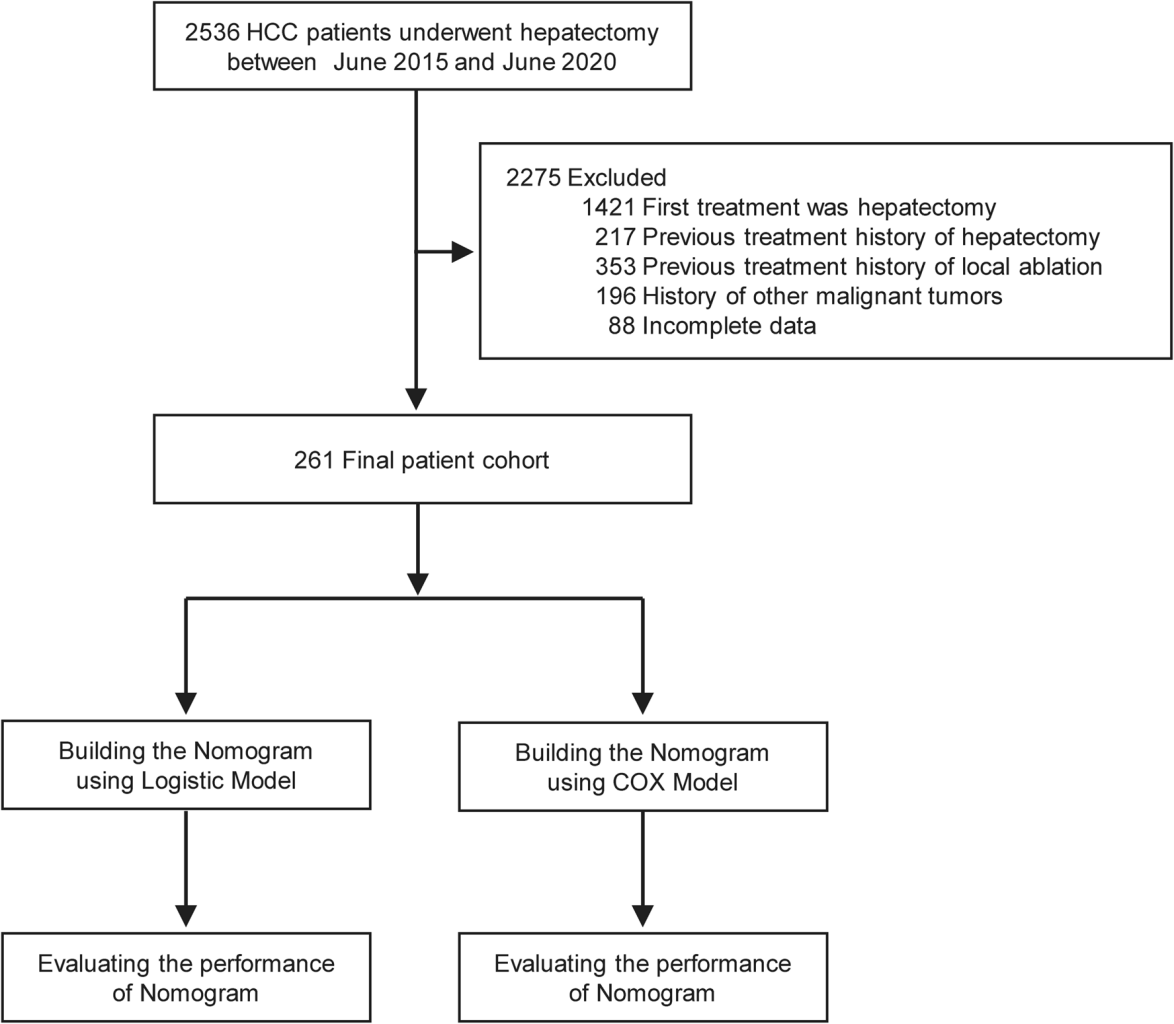
Flow diagram of the study enrollment patients. HCC, hepatocellular carcinoma

During the study period, a total of 261 HCC patients who underwent conversion hepatectomy and were previously treated with transarterial interventional therapy were enrolled (the inclusion criteria are shown in Fig. 1).

In total, 47, 151, and 63 patients received TACE, HAIC, and TACE combined with HAIC, respectively. At a median follow-up of 16.8 months (range 1.1–61.8 months), 54.8% (143 of 261) of the patients had recurrence. The median RFS was 9.5 months (95% CI 7.8–10.8 months). The 1-, 2-, and 3-year RFS percentages were 38.7%, 12.3%, and 6.1%, respectively. The clinicopathologic characteristics of the patients are provided in Table 1 and Additional file 4: Table S1.

**Table 1:** Participant characteristics

Variable	No. (%) of entire population (*n* = 261)
Age, years, median (IQR)	61 (16–79)
< 65 years	223 (85.4)
≥ 65 years	38 (14.6)
Gender (male/female)	
Male	218 (83.5)
Female	43 (16.5)
Tumor size	
≤ 3 cm	7 (2.7)
3–5 cm	34 (13)
≥ 5 cm	220 (84.3)
Previous intervention therapy	
TACE	47 (18)
HAIC	151 (57.9)
TACE + HAIC	63 (24.1)
Tumor number	
Solitary	119 (45.6)
Multiple	142 (54.4)
MVI	
0	173 (66.3)
1	51 (19.5)
2	37 (14.2)
BCLC stage	
A	98 (37.5)
B	71 (27.2)
C	92 (35.3)
*Preintervention serum tests*	
Platelets (10^3^/mm^3^)	
100–300	200 (76.6)
< 100	6 (2.3)
> 300	55 (21.1)
INR	
0.85–1.2	247 (94.6)
> 1.2	14 (5.4)
AFP (ng/mL)	
< 400	113 (43.3)
≥ 400	148 (56.7)
PIVKA-II (mAU/mL)	
< 40	13 (5)
≥ 40	248 (95)
CA19-9 (U/mL)	
≤ 35	177 (67.8)
> 35	84 (32.2)
ALB (g/L)	
> 35	254 (97.3)
≤ 35	7 (2.7)
TBIL (μmol/L)	
≤ 20.5	226 (86.6)
> 20.5	35 (13.4)
*Preoperative serum tests*	
Platelets (10^3^/mm^3^)	
100–300	229 (87.7)
< 100	32 (12.3)
INR	
0.85–1.2	256 (98.1)
> 1.2	5 (1.9)
AFP (ng/mL)	
< 400	176 (67.4)
≥ 400	85 (32.6)
PIVKA-II (mAU/mL)	
< 40	66 (25.3)
≥ 40	195 (74.7)
CA19-9 (U/mL)	
≤ 35	179 (68.6)
> 35	82 (31.4)
ALB (g/L)	
> 35	249 (95.4)
≤ 35	12 (4.6)
TBIL (μmol/L)	
≤ 20.5	249 (95.4)
> 20.5	12 (4.6)
*Postoperative serum tests*	
Platelets (10^3^/mm^3^)	
100–300	230 (88.5)
< 100	31 (11.5)
INR	
0.85–1.2	257 (98.5)
> 1.2	4 (1.5)
AFP (ng/mL)	
< 400	232 (88.9)
≥ 400	29 (11.1)
PIVKA-II (mAU/mL)	
< 40	201 (77)
≥ 40	60 (23)
CA19-9 (U/mL)	
≤ 35	194 (74.3)
> 35	67 (25.7)
ALB (g/L)	
> 35	253 (96.9)
≤ 35	8 (3.1)
TBIL (μmol/L)	
≤ 20.5	245 (93.9)
> 20.5	16 (6.1)
ECOG PS	
0	200 (76.6)
1	61 (23.4)

AFP, alpha‐fetoprotein; ALB, albumin; BCLC, Barcelona‐Clinic Liver Cancer; CA19-9, carbohydrate antigen 19-9; ECOG PS, Eastern Cooperative Oncology Group performance score; HAIC, hepatic artery infusion chemotherapy; INR, international normalized ratio; IQR, interquartile range; MVI, microvascular invasion; PIVKA-II, protein induced by vitamin K absence or antagonist-II; TACE, transcatheter arterial chemoembolization; TBIL, total bilirubin

### Model specifications and independent prognostic factors

According to univariable logistic regression analysis, tumor number (HR, 1.699; 95% CI 1.016–2.985), microvascular invasion (MVI) grade (for grade 1 vs. grade 0, HR, 2.104; 95% CI 1.082–4.51; for grade 2 vs. grade 0, HR, 2.864; 95% CI 1.235–6.644), preoperative alpha‐fetoprotein (AFP) level (HR, 2.848; 95% CI 1.501–5.406), preoperative carbohydrate antigen 19-9 (CA19-9) level (HR, 3.653; 95% CI 1.723–7.746), and Eastern Cooperative Oncology Group performance score (ECOG PS) (HR, 3.032; 95% CI 1.471–6.250) increased the risk of recurrence. Conversely, age (3.002; 95% CI 1.299–6.938) decreased this risk (Table 2). These variables were included in a multivariable logistic model. Different transarterial interventional therapies in this study did not show statistically significant (Additional files 5, 6: Table S2, S3, and Additional file 1: Fig. S1).

**Table 2:** Logistic regression model showing the association of variables with the probability of recurrence

Variable	Univariate analysis		Multivariate analysis	
	OR (95% CI)	*p-*value	OR (95% CI)	*p-*value
Age, years, < 65 versus ≥ 65	2.962 (1.146–2.499)	0.01	3.002 (1.299–6.938)	0.01
Tumor number, multiple versus solitary	1.568 (0.959–2.564)	0.073	1.699 (1.016–2.985)	0.048
MVI				
1 versus 0	2.748 (1.278–5.909)	0.01	2.104 (1.082–4.51)	0.046
2 versus 0	3.072 (1.551–6.087)	0.001	2.864 (1.235–6.644)	0.014
Preintervention CA199, > 35 versus ≤ 35 U/mL	1.428 (0.842–2.421)	0.186	0.64 (0.309–1.322)	0.227
Preoperative AFP, ≥ 400 versus < 400 ng/mL	2.914 (1.669–5.088)	< 0.001	2.848 (1.501–5.406)	0.001
Preoperative PIVKA-II, ≥ 40 versus < 40 mAU/mL	1.84 (1.123–3.013)	0.015	1.559 (0.88–2.763)	0.128
Preoperative CA19-9, > 35 versus ≤ 35 U/mL	2.466 (1.417–4.291)	0.001	3.653 (1.723–7.746)	0.001
ECOG PS, score 1 versus score 0	1.97 (1.079–3.597)	0.003	3.032 (1.471–6.250)	0.003

AFP, alpha‐fetoprotein; CA19-9, carbohydrate antigen 19-9; ECOG PS, Eastern Cooperative Oncology Group performance score; MVI, microvascular invasion; PIVKA-II, protein induced by vitamin K absence or antagonist-II

The predictors of RFS in univariate analysis are listed in Table 3. All significant factors in univariate analysis were included in multivariate Cox regression analysis. Five predictive factors, including age (HR, 2.615; 95% CI 1.433–4.773), tumor number (HR, 1.532; 95% CI 1.074–2.185), postoperative AFP level (for 25–400 vs. ≤ 25, HR, 2.597; 95% CI 1.589–4.246; for ≥ 400 vs. ≤ 25, HR, 3.222; 95% CI 1.683–6.167), postoperative protein induced by vitamin K absence or antagonist-II (PIVKA-II) level (HR, 1.739; 95% CI 1.166–2.593), and ECOG PS (HR, 1.523; 95% CI 1.026–2.261), were adopted in the final model.

**Table 3:** Cox proportional hazards regression model showing the association of variables with the recurrence-free survival

Variable	Univariate analysis		Multivariate analysis	
	OR (95% CI)	*p-*value	OR (95% CI)	*p-*value
Age, years, < 65 versus ≥ 65	2.244 (1.291–3.899)	0.004	2.615 (1.433–4.773)	0.002
Tumor number, multiple versus solitary	1.48 (1.057–2.072)	0.022	1.532 (1.074–2.185)	0.019
MVI				
1 versus 0	1.726 (1.108–2.69)	0.016	1.282 (0.845–1.945)	0.243
2 versus 0	1.908 (1.29–2.822)	0.001	1.511 (0.934–2.443)	0.092
Preintervention INR	1.996 (0.976–4.084)	0.058	1.781 (0.801–3.957)	0.157
Preintervention CA19-9, > 35 versus ≤ 35 U/mL	1.333 (0.946–1.879)	0.101	0.814 (0.525–1.264)	0.36
Preoperative AFP, ≥ 400 versus < 400 ng/mL	1.8 (1.291–2.511)	0.001	0.845 (0.535–1.334)	0.469
Preoperative CA19-9, > 35 versus ≤ 35 U/mL	1.537 (1.098–2.15)	0.012	1.332 (0.853–2.078)	0.207
Postoperative AFP, ng/mL				
25–400 versus ≤ 25	2.816 (1.918–4.135)	< 0.001	2.597 (1.589–4.246)	< 0.001
≥ 400 versus ≤ 25	3.172 (1.924–5.231)	< 0.001	3.222 (1.683–6.167)	< 0.001
Postoperative PIVKA-II, > 40 versus ≤ 40 mAU/mL	2.37 (1.649–3.406)	< 0.001	1.739 (1.166–2.593)	0.007
ECOG PS, score 1 versus score 0	1.514 (1.049–2.186)	0.027	1.523 (1.026–2.261)	0.037

AFP, alpha‐fetoprotein; CA19-9, carbohydrate antigen 19-9; ECOG PS, Eastern Cooperative Oncology Group performance score; INR, international normalized ratio; MVI, microvascular invasion; PIVKA-II, protein induced by vitamin K absence or antagonist-II

### Nomograms and model performance

**Fig. 2:**
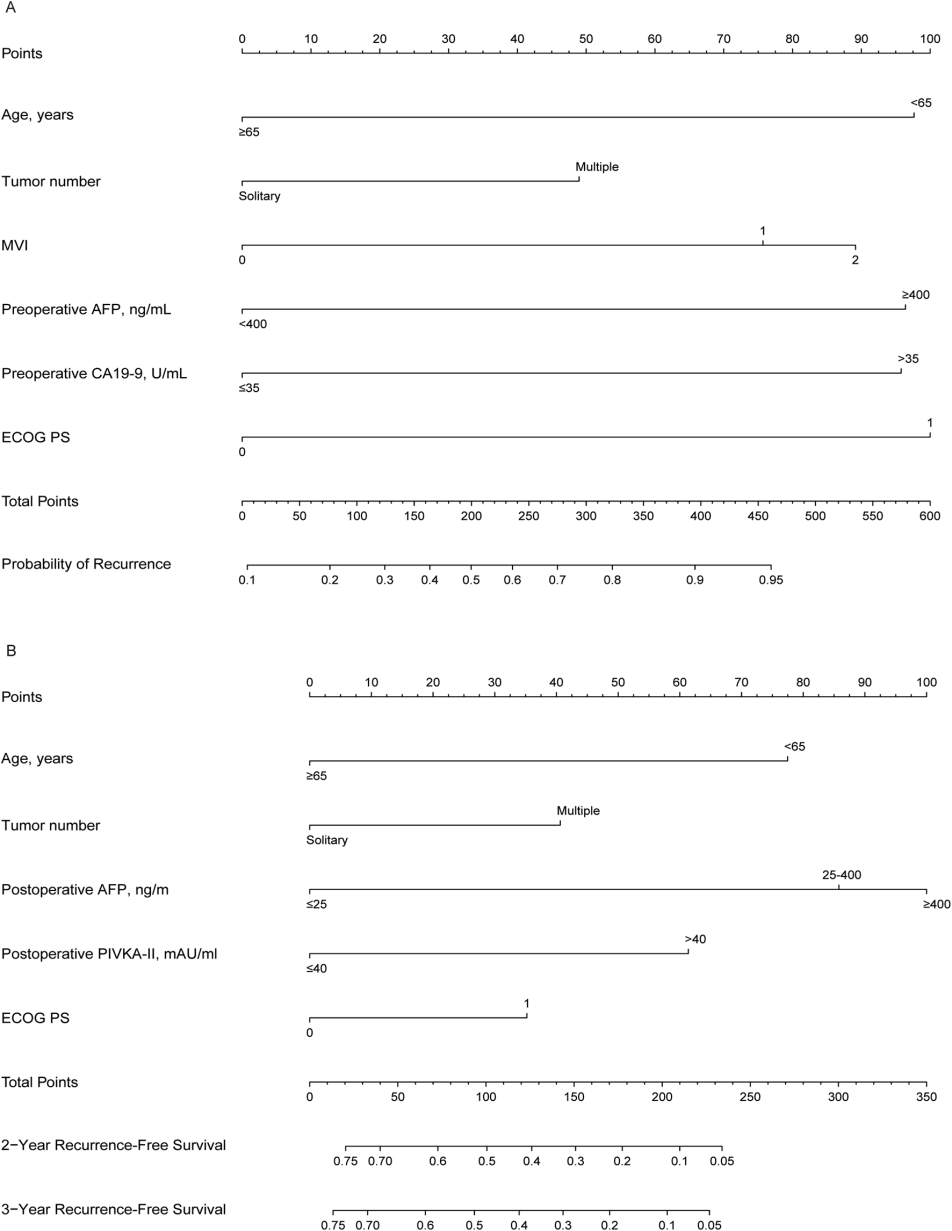
Nomograms for predicting recurrence and recurrence-free survival. **A** The nomogram to predict the probability of recurrence was generated based on 6 independent prognostic factors. **B** The nomogram to predict recurrence-free survival was created based on 5 independent prognostic factors (see the “Model specifications and independent prognostic factors” section of the “Results” section)

Nomograms to predict the probability of recurrence and RFS of patients treated with conversion liver resection and previously received transarterial interventional therapy are depicted in Fig. 2. The nomogram to predict the probability of recurrence was generated based on the following six independent prognostic factors: age, tumor number, MVI grade, preoperative AFP level, preoperative CA19-9 level, and ECOG PS. The nomogram to predict RFS was built using five independent prognostic factors, namely, age, tumor number, postoperative AFP level, postoperative PIVKA-II level, and ECOG PS.

Patients with a higher score had a higher probability of recurrence or low RFS. For example, a case of a 50-year-old patient with a solitary tumor, MVI grade 1, preoperative AFP ≥ 400 ng/mL, preoperative CA19-9 > 35 U/mL, and ECOG PS equal to 0 would score a total of 320 points (40 points for age, 0 points for tumor number, 82.5 points for MVI, 97.5 points for preoperative AFP, 100 points for preoperative CA19-9, and 0 points for ECOG PS), for a probability of recurrence of 85%. Similarly, a case of a 65-year-old patient with multiple tumors, postoperative AFP ≤ 25 ng/m, postoperative PIVKA-II > 40 mAU/mL, and ECOG PS equal to 1 would have a total of 137.5 points (0 points for age, 40 points for tumor number, 0 points for postoperative AFP, 62.5 points for postoperative PIVKA-II, and 35 points for ECOG PS). For this case, the predicted probability of 2-year and 3-year RFS was 35.0% and 31.0%, respectively.

The bootstrap validation method was performed for internal validation. The nomograms demonstrated good accuracy in estimating the probability of recurrence and RFS, with an unadjusted C-index of 0.758 (95% CI 0.7–0.817) and a bootstrap-corrected C-index of 0.749 (95% CI 0.704–0.814) for the probability of recurrence. The C-index for RFS prediction was 0.701 (95% CI 0.654–0.748). The areas under the ROC curve (AUCs) for the 12-, 24, 36-, and 48-month RFS were 0.736 (95% CI 65.18–78.72), 0.735 (95% CI 62.94–79.05), 0.711 (95% CI 58.49–78.96), and 0.702 (95% CI 55.75–81.87), respectively. In addition, the median nomogram score was used to divide the patients into the low-risk and high-risk groups. The Kaplan–Meier method was performed to analyze the RFS. As a result, the RFS survival probability of the high-risk group was significantly lower than that of the low-risk group in the whole cohort. RFS analysis was performed using the same method based on the AJCC8th and BCLC staging systems.

**Fig. 3:**
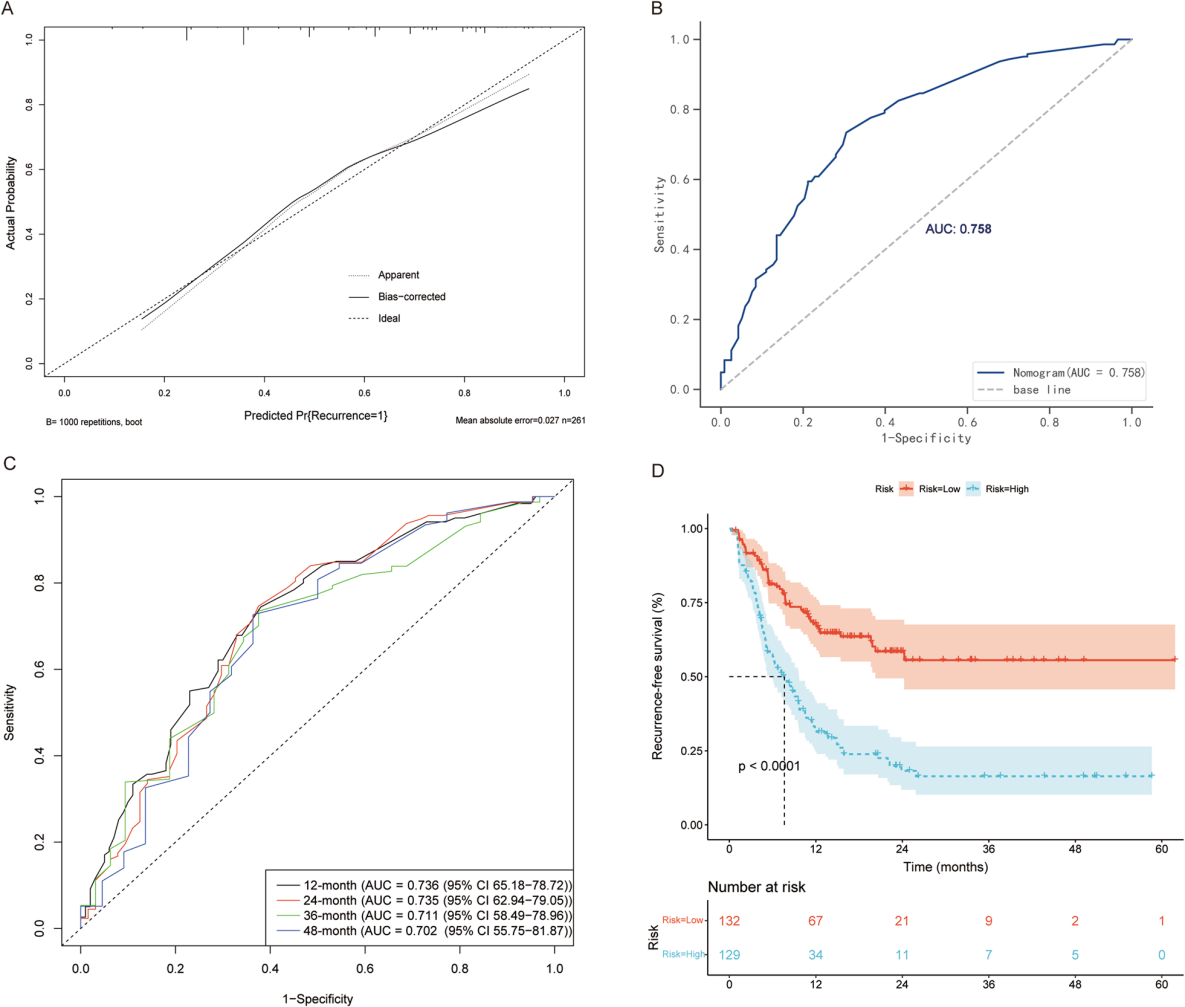
Model performance. **A** Calibration plot comparing predicted and actual probability of recurrence. **B** Receiver operator characteristic (ROC) curve and the corresponding area of the predictive model for the probability of recurrence. **C** ROC curves evaluating predictive performance of the nomogram for 12, 24, 36, and 48 months in the cohort. **D** Recurrence-free survival was compared between patients with nomogram the low-risk and the high-risk scores in the cohort

Similarly, the RFS survival rate of the low-risk group was significantly better than that of the high-risk group (Additional file 2: Fig. S2). Moreover, the calibration plots graphically showed good agreement between the prediction by the nomograms and the actual observation. The Kaplan–Meier curve and the calibration plots are illustrated in Fig. 3 and Additional file 3: Fig. S3.

## Discussion

Although radical resection is still the preferred treatment for HCC, the risk of postoperative recurrence is high, with more than 50% of patients experiencing recurrence [14]. Similarly, 54.8% (143 of 261) of patients in our research had disease recurrence. Our findings also suggested that age, tumor number, MVI, preoperative AFP level, preoperative CA19-9 level, and ECOG PS score are significantly associated with recurrence in HCC patients who undergo conversion hepatectomy and were previously treated with transarterial interventional therapy.

Two nomograms to predict a patient’s recurrence probability and RFS after conversion hepatectomy for HCC based on clinicopathological parameters were developed in this study. According to the median nomogram score, patients in the low-risk group have a better RFS rate than those in the high-risk group. Our results can be used to predict the recurrence risk of patients and to develop individualized strategies for treatment and surveillance for those with high recurrence rates. The current research is significant because predictive models and nomograms were for the first time generated for HCC patients treated with conversion liver resection and previously underwent transarterial interventional therapy.

Another notable advantage of this study is that previously reported essential variables related to the outcomes after hepatectomy were taken into account [15–17]. Although previous studies [15, 18, 19] have attempted to predict HCC patient recurrence by clinicopathological features, the factors reported to be related to outcomes vary widely. AFP is a key tumor marker of HCC, and it is also included as a factor in many prognosis models for HCC patients [8, 20]. PIVKA-II has also been applied as a tumor marker for HCC in recent years [21, 22], and it can be used as a complement to AFP to improve HCC diagnosis [8]. CA19-9 is often utilized to diagnose cholangiocarcinoma or pancreatic cancer [23–26]. Nevertheless, recent studies have shown that abnormal levels of serum CA19-9 have diagnostic value for the prognosis of patients with HCC [27, 28]. Our study verified these conclusions. Other studies have reported [17, 18, 29–33] that AFP, PIVKA-II, and CA19-9 levels, vascular invasion, tumor number, MVI, and age are closely associated with the risk of recurrence, and another study [34] revealed an association between ECOG PS and poor RFS in patients with HCC. Nevertheless, it has been demonstrated [19, 35, 36] that age, sex, AFP, PIVKA-II, tumor number, or ECOG PS have little association with outcome. Some studies [37, 38] have reported prediction models incorporating molecular and serum markers or systemic immune inflammation and prognostic nutritional indices for estimating recurrence after liver resection. However, these parameters are not easy to measure, and some parameters recognized as critical for tumor recurrence were not considered in the predictive model. Moreover, some factors in this model require specific detection methods, and are thus not widely used. In general, easy-to-use predictive tools are needed for clinical work. Nomograms are not only convenient to use but also have high accuracy and good discrimination characteristics in terms of predicting results [11]. In the present research, the nomograms constructed contained variables that are comprehensive and easy to obtain. The C-index value of the recurrence prediction model was 0.758, and the C-index values of the RFS prognostic model were 0.736, 0.735, 0.711, and 0.702 for 1, 2, 3, and 4 years, respectively. The optimal calibration curve showed that the predicted value was consistent with the observed value.

At the time of their first visit, approximately 80% of patients with HCC have lost the opportunity to receive radical liver resection due to a large tumor size, multiple nodules, or vascular invasion [9]. At present, surgical resection remains a critical therapeutic strategy for HCC patients, offering the possibility of long-term survival. Conversion treatment is a kind of nonsurgical treatment for patients who at the first visit are not suitable for surgical resection, such as interventional therapy [6]. After treatment, a certain degree of tumor downstaging can be achieved to allow the opportunity for radical resection [7]. Indeed, conversion surgery is of great significance to the treatment of tumor patients, and it is a hot research topic.

In this study, we retrospectively analyzed patients who had received interventional therapy and were not suitable for surgery at their first visit. By undergoing interventional treatment, such as TACE, HAIC, or TACE + HAIC, these HCC patients were successfully treated with conversion radical hepatectomy. However, high tumor recurrence after surgery is still a challenge for achieving long-term survival in HCC. We constructed nomograms for predicting recurrence risk and RFS by analyzing recurrence of HCC patients undergoing liver resection. The nomograms have important guiding significance for clinical treatment. In general, postoperative monitoring and follow-up should be performed for patients who have undergone interventional conversion therapy and are prone to recurrence after surgery. Adjuvant treatment should also be considered after surgery according to the patient’s high-risk factors, such as MVI, multiple tumors, and high ECOG PS scores. Additionally, the interval between re-examination cycles should be short, and adjuvant therapies should be performed individually following the operation.

The current study had several limitations. First, this research was a single-institution retrospective analysis. Although the proposed nomograms had good C-indices of 0.758 and 0.711, future large-sample, multi-center, and prospective verification are needed to validate the proposed nomograms externally. Second, the sample size was small, and some analyses may have been limited. Since HAIC has gradually been used for HCC treatment in some areas in recent years, and fewer patients can be successfully converted to surgical resection after HAIC or TACE, a certain amount of time will be required to accumulate more sample size of conversion resection and multi-center external validation. Third, as with all surgical retrospective studies, selection bias may be present. Finally, the models were generated according to clinicopathological parameters, and more specific markers need to be explored to improve the accuracy of tumor recurrence and RFS prediction.

## Conclusions

In conclusion, we developed reliable two nomograms based on single-center data for HCC patients who underwent conversion liver resection and were previously treated with transarterial interventional therapy. Several independent prognostic factors were identified to predict the risk of recurrence and RFS. These results are helpful for predicting the risk of HCC recurrence and the probability of RFS and will act as a guide for surgeons with regard to the choice of therapeutic strategy for HCC patients. In order to increase the reliability, the next research direction of our team is preparing for prospective multi-center research. Future studies should verify the proposed nomograms externally to prove their value in clinical prognosis prediction for HCC patients after conversion hepatectomy.

### Supplementary Information

**Additional file 1. Fig. S1**: Kaplan–Meier curves for the recurrence-free survival of patients receiving transcatheter arterial chemoembolization (TACE), hepatic artery infusion chemotherapy (HAIC), and TACE combined with HAIC.

**Additional file 2. Fig. S2**: Kaplan–Meier survival curves of RFS for BCLC (**A**) and AJCC 8th/TNM (**B**) staging systems.

**Additional file 3. Fig. S3**: Calibration plots comparing predicted and actual recurrence-free survival probabilities at 12 (**A**), 24 (**B**), 36 (**C**), and 48 months (**D**) of follow-up.

**Additional file 4. Table S1**: Patient serum tests.

**Additional file 5. Table S2**: Logistic regression model showing the types of transarterial interventional therapy with the probability of recurrence.

**Additional file 6. Table S3**: Cox proportional hazards regression model showing the types of transarterial interventional therapy with the recurrence-free survival.

## Data Availability

The data that support the findings of our study are available upon request from the corresponding author. The data are not publicly available due to privacy or ethical restrictions.

## Abbreviations

AFP: Alpha‐fetoprotein
AUROC: Area under the Receiver Operator Characteristic
CA19-9: Carbohydrate antigen 19-9
C-index: Concordance index
CR: Complete response
CT: Computed tomography CT
ECOG PS: Eastern Cooperative Oncology Group performance score ECOG PS
HAIC: Hepatic artery infusion chemotherapy
HCC: Hepatocellular carcinoma
H–L: Hosmer–Lemeshow
IQR: Interquartile range
mRECIST: Modified Response Evaluation Criteria in Solid Tumors
MRI: Magnetic resonance imaging
MVI: Microvascular invasion
PD: Progressive disease
PIVKA-II: Protein induced by vitamin K absence or antagonist-II
PR: Partial response
RFS: Recurrence-free survival
SD: Stable disease
TACE: Transcatheter arterial chemoembolization
