# Supplementary figures and images for: Development of nomograms to predict recurrence after conversion hepatectomy for hepatocellular carcinoma previously treated with transarterial interventional therapy
Source: Eur J Med Res. 2023 Sep 9;28:328. doi: 10.1186/s40001-023-01310-4 (PMC10492285; doi:10.1186/s40001-023-01310-4)

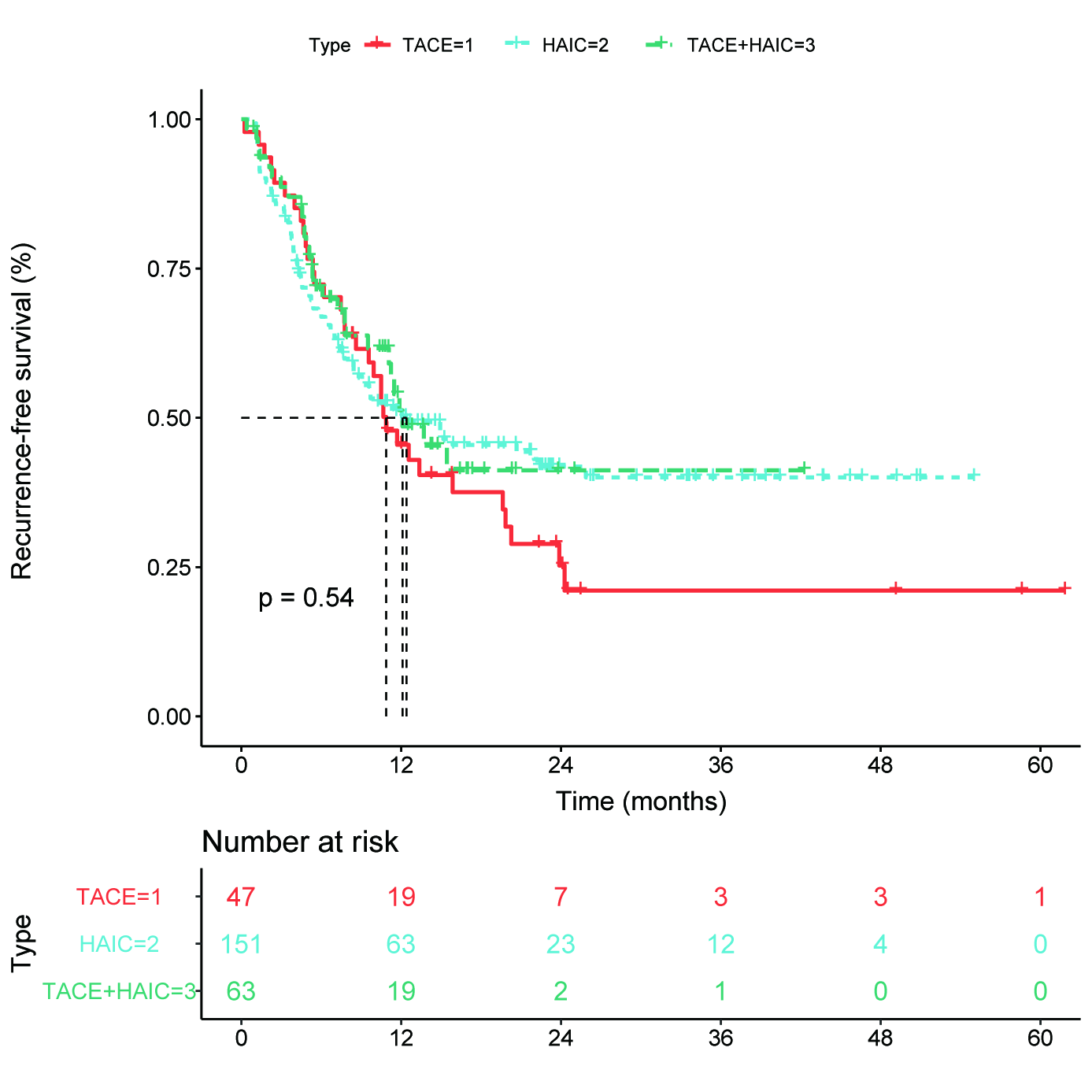

Supplement: Supplementary file 1 — Additional file 1. Fig. S1: Kaplan–Meier curves for the recurrence-free survival of patients receiving transcatheter arterial chemoembolization (TACE), hepatic artery infusion chemotherapy (HAIC), and TACE combined with HAIC. [file 40001_2023_1310_MOESM1_ESM.tif]

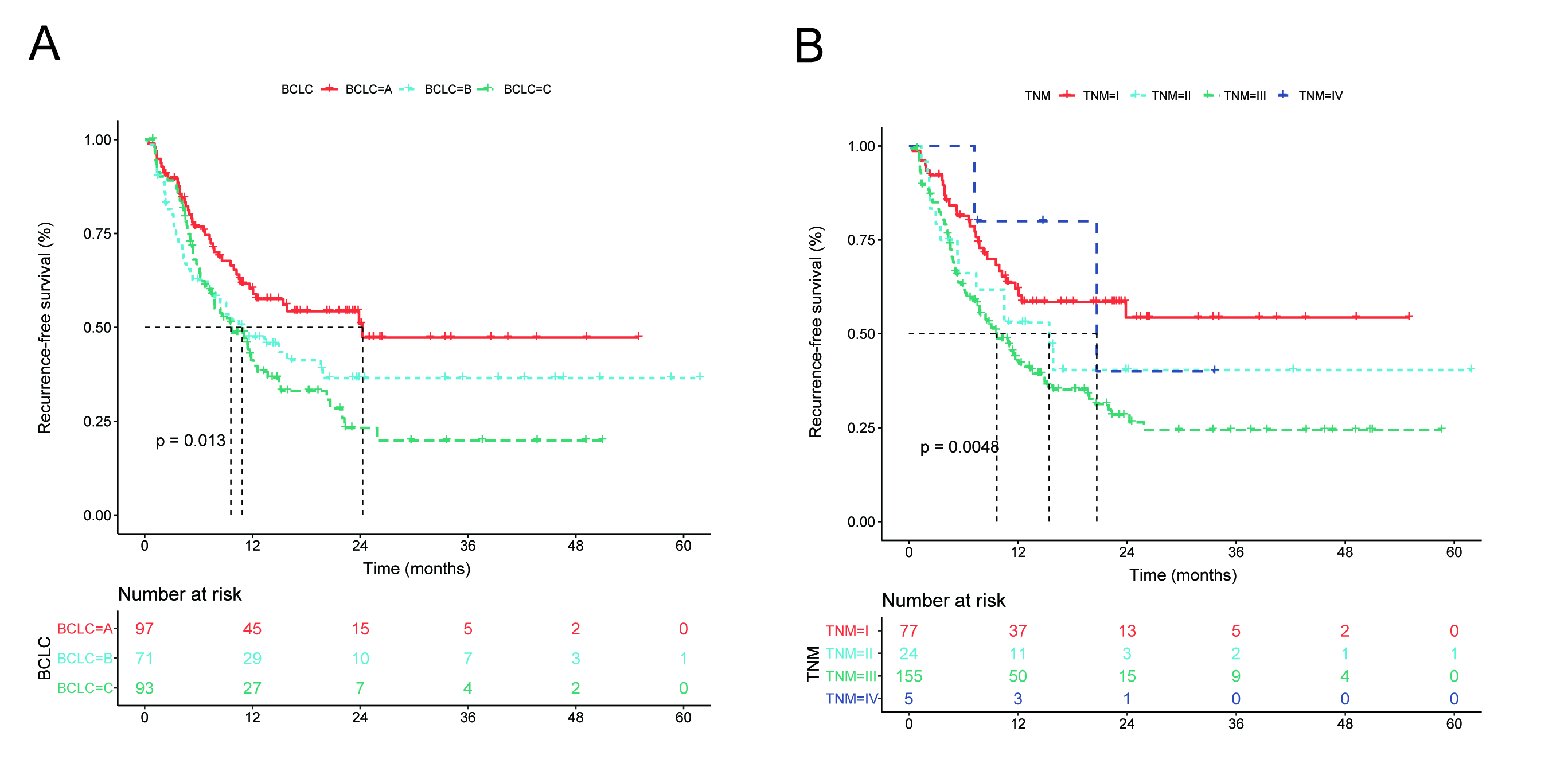

Supplement: Supplementary file 2 — Additional file 2. Fig. S2: Kaplan–Meier survival curves of RFS for BCLC (A) and AJCC 8th/TNM (B) staging systems. [file 40001_2023_1310_MOESM2_ESM.tif]

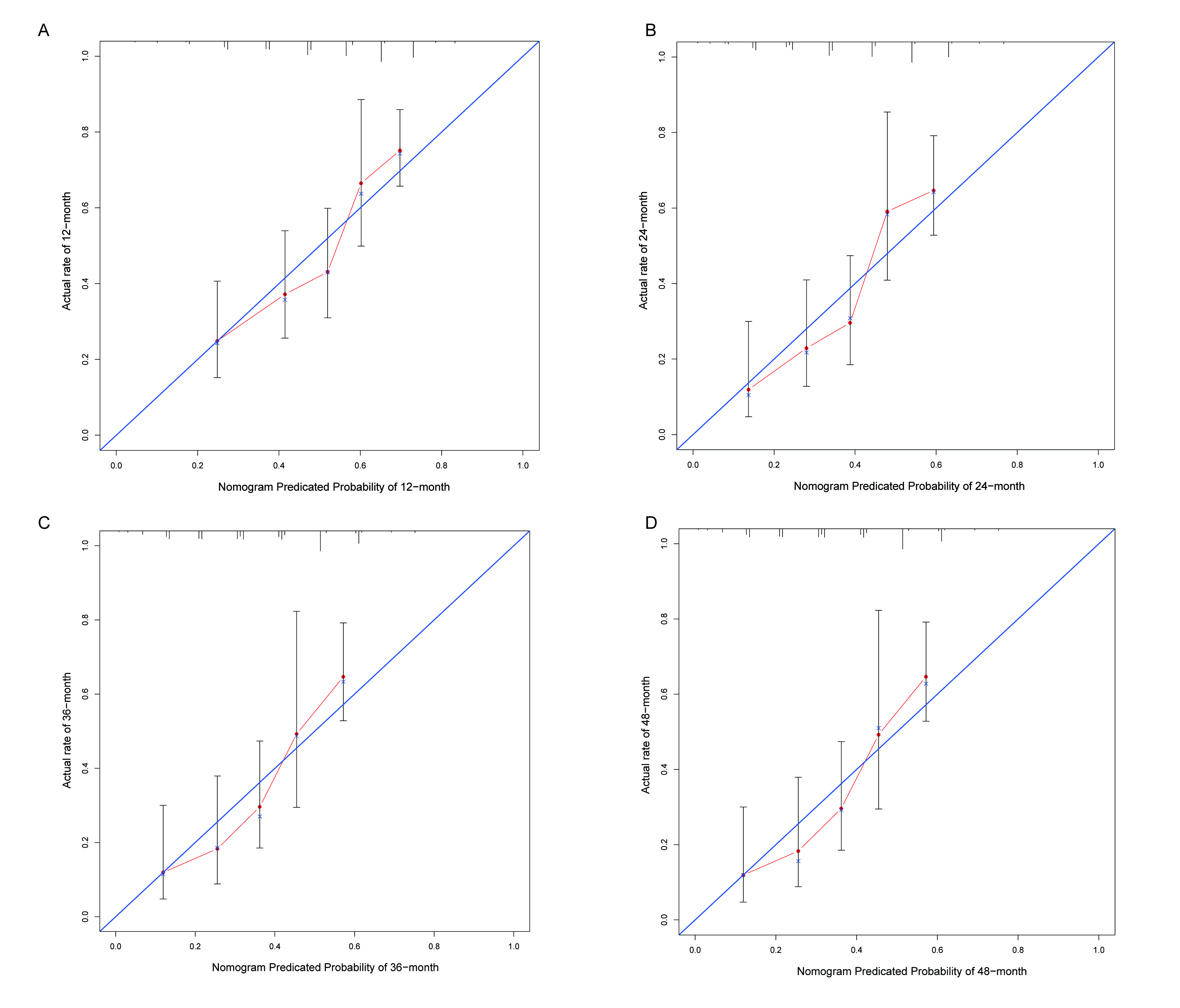

Supplement: Supplementary file 3 — Additional file 3. Fig. S3: Calibration plots comparing predicted and actual recurrence-free survival probabilities at 12 (A), 24 (B), 36 (C), and 48 months (D) of follow-up. [file 40001_2023_1310_MOESM3_ESM.tif]
