# Supplementary material for: Development of nomograms to predict recurrence after conversion hepatectomy for hepatocellular carcinoma previously treated with transarterial interventional therapy
Source: Eur J Med Res. 2023 Sep 9;28:328. doi: 10.1186/s40001-023-01310-4 (PMC10492285; doi:10.1186/s40001-023-01310-4)
Supplement: Supplementary file 4 — Additional file 4. Table S1: Patient serum tests. [file 40001_2023_1310_MOESM4_ESM.docx]

**Table S1. Patient Serum Tests**

| **Variable** | **No. (%) of Entire Population (n=261)** |
| --- | --- |
| **Preintervention serum tests** |  |
| Platelets (10^3^/mm^3^) |  |
| 100-300 | 200 (76.6) |
| ＜100 | 6 (2.3) |
| ＞300 | 55 (21.1) |
| INR |  |
| 0.85-1.2 | 247 (94.6) |
| ＞1.2 | 14 (5.4) |
| AFP (ng/mL) |  |
| ＜400 | 113 (43.3) |
| ≥400 | 148 (56.7) |
| PIVKA-II (mAU/ml) |  |
| ＜40 | 13 (5) |
| ≥40 | 248 (95) |
| CA19-9 (U/mL) |  |
| ≤35 | 177 (67.8) |
| ＞35 | 84 (32.2) |
| ALB (g/L) |  |
| ＞35 | 254 (97.3) |
| ≤35 | 7 (2.7) |
| TBIL (μmol/L) |  |
| ≤20.5 | 226 (86.6) |
| >20.5 | 35 (13.4) |
| **Preoperative** **serum tests** |  |
| Platelets (10^3^/mm^3^) |  |
| 100-300 | 229 (87.7) |
| ＜100 | 32 (12.3) |
| INR |  |
| 0.85-1.2 | 256 (98.1) |
| ＞1.2 | 5 (1.9) |
| AFP (ng/mL) |  |
| ＜400 | 176 (67.4) |
| ≥400 | 85 (32.6) |
| PIVKA-II (mAU/ml) |  |
| ＜40 | 66 (25.3) |
| ≥40 | 195 (74.7) |
| CA19-9 (U/mL) |  |
| ≤35 | 179 (68.6) |
| ＞35 | 82 (31.4) |
| ALB (g/L) |  |
| ＞35 | 249 (95.4) |
| ≤35 | 12 (4.6) |
| TBIL (μmol/L) |  |
| ≤20.5 | 249 (95.4) |
| >20.5 | 12 (4.6) |
| **Postoperative serum tests** |  |
| Platelets (10^3^/mm^3^) |  |
| 100-300 | 230 (88.5) |
| ＜100 | 31 (11.5) |
| INR |  |
| 0.85-1.2 | 257 (98.5) |
| ＞1.2 | 4 (1.5) |
| AFP (ng/mL) |  |
| ＜400 | 232 (88.9) |
| ≥400 | 29 (11.1) |
| PIVKA-II (mAU/ml) |  |
| ＜40 | 201 (77) |
| ≥40 | 60 (23) |
| CA19-9 (U/mL) |  |
| ≤35 | 194 (74.3) |
| ＞35 | 67 (25.7) |
| ALB (g/L) |  |
| ＞35 | 253 (96.9) |
| ≤35 | 8 (3.1) |
| TBIL (μmol/L) |  |
| ≤20.5 | 245 (93.9) |
| >20.5 | 16 (6.1) |

Abbreviations: AFP, alpha‐fetoprotein; ALB, albumin; CA19-9, carbohydrate antigen 19-9; INR, international normalized ratio; PIVKA-II, protein induced by vitamin K absence or antagonist-II; TBIL, total bilirubin.
