# Supplementary material for: Development of nomograms to predict recurrence after conversion hepatectomy for hepatocellular carcinoma previously treated with transarterial interventional therapy
Source: Eur J Med Res. 2023 Sep 9;28:328. doi: 10.1186/s40001-023-01310-4 (PMC10492285; doi:10.1186/s40001-023-01310-4)
Supplement: Supplementary file 5 — Additional file 5. Table S2: Logistic regression model showing the types of transarterial interventional therapy with the probability of recurrence. [file 40001_2023_1310_MOESM5_ESM.docx]

**Table S2 Logistic Regression Model Showing the Types of Transarterial Interventional Therapy with the Probability of Recurrence**

|  | Univariable Analysis |  | Multivariable Analysis* |  |
| --- | --- | --- | --- | --- |
| Variable | OR (95% CI) | *p-*Value | OR (95% CI) | *p-*Value |
| TACE | 1.063 (0.590-1.914) | 0.839 | - | - |
| HAIC | 2.067 (0.94-4.542) | 0.071 | - | - |
| TACE+HAIC | 1.032 (0.612-1.848) | 0.133 | - | - |

* Since there was no statistical difference in univariate analysis, these factors were not included in multivariate analysis.

Abbreviations: HAIC, hepatic artery infusion chemotherapy; TACE, transcatheter arterial chemoembolization.
