# Supplementary material for: Development of nomograms to predict recurrence after conversion hepatectomy for hepatocellular carcinoma previously treated with transarterial interventional therapy
Source: Eur J Med Res. 2023 Sep 9;28:328. doi: 10.1186/s40001-023-01310-4 (PMC10492285; doi:10.1186/s40001-023-01310-4)
Supplement: Supplementary file 6 — Additional file 6. Table S3: Cox proportional hazards regression model showing the types of transarterial interventional therapy with the recurrence-free survival. [file 40001_2023_1310_MOESM6_ESM.docx]

**Table S3 Cox Proportional Hazards Regression Model Showing the Types of Transarterial Interventional Therapy with the Recurrence-Free Survival**

|  | Univariable Analysis |  | Multivariable Analysis* |  |
| --- | --- | --- | --- | --- |
| Variable | OR (95% CI) | *p-*Value | OR (95% CI) | *p-*Value |
| TACE | 1.126 (0.689-1.841) | 0.635 | - | - |
| HAIC | 0.922 (0.609-1.395) | 0.7 | - | - |
| TACE+HAIC | 0.924 (0.667-1.542) | 0.631 | - | - |

* Since there was no statistical difference in univariate analysis, these factors were not included in multivariate analysis.

Abbreviations: Abbreviations: HAIC, hepatic artery infusion chemotherapy; TACE, transcatheter arterial chemoembolization.
